# Influence of infarct healing on left ventricular remodeling in patients with acute st-segment elevation myocardial infarction

**DOI:** 10.1186/1532-429X-13-S1-P135

**Published:** 2011-02-02

**Authors:** Pier Giorgio Masci, Anca Floran, Vincenzo Positano, Steven Dymarkowski, Walter Desmet, Massimo Lombardi, Stefan Janssens, Jan Bogaert

**Affiliations:** 1 Fondazione Toscana/CNR "G. Monasterio", Pisa, Italy; 2 Gasthuisberg University Hospital, Leuven, Belgium

## Background

Infarct healing is a complex process consisting in replacement of necrotic tissue with fibrotic scar. Animal studies indicate that this process is highly anisotropic showing different patterns, which may ultimately influence left ventricular (LV) remodeling.

Study aim: to investigate the influence of infarct healing on post-infarction LV remodeling in patients with acute ST-segment elevation myocardial infarction (MI).

## Methods

Seventy-eight patients with acute ST-segment elevation MI treated by percutaneous coronary intervention within 12-hour from symptoms onset were studied by cardiovascular magnetic resonance (CMR) within one-week (baseline) and at four-month (follow-up) after MI. Cine CMR was used to assess LV volumes, mass and function. Early and late post-gadolinium 3-dimensional T1-weighted inversion-recovery segmented gradient-echo CMR in short and long-axis directions was utilized to depict microvascular obstruction (MO) and myocardial necrosis/fibrosis, respectively. On late post-contrast short-axis images, the following parameters were measured: infarct mass, infarct-wall thickness, infarct-thickness (ie, radial extent of infarction), infarct transmurality and circumferential infarct length (CIL). Longitudinal infarct length (LIL) was derived on long-axis late post-contrast images. Infarct-surface was calculated multiplying CIL by slice thickness (ie, 5 mm).

## Results

Infarct size was 25±19g (19±12% of LV) at baseline and 13±9g (12±8% of LV) at follow-up (p<0.001), yielding an infarct size reduction of 45±17%. Non-infarcted myocardium tended to decrease during follow-up (p=0.051). Infarct resorption was more pronounced in the radial direction (31±15%) than circumferential (10±14%) or longitudinal (10±11%) ones (p<0.001). Infarct-surface reduction was 10±16% (range -26% to 53%), and 14 patients increased infarct-surface during follow-up (-12±6%) indicating chronic infarct expansion. Infarct-wall thinning was positively and strongly related to the infarct-thickness reduction (r=0.76, p<0.001) but not to overall MI shrinkage or to infarct-surface reduction. At multivariable linear regression analysis, the increase of infarct-surface during follow-up was independently associated to increase of LV end-diastolic volume during follow-up (Beta-coefficient=0.358, p=0.020) even after correction for baseline infarct size, MO extent, infarct-thickness reduction and LIL reduction.

## Conclusions

Infarct healing is an anisotropic process occurring preferentially along the radial direction and playing a crucial role in post-infarction LV remodeling. Indeed, the degree of infarct radial resorption is strongly related to the infarct-wall thinning whereas the increase of infarct surface during follow-up (ie, chronic infarct expansion) is an independent predictor of adverse LV remodeling.

